# Dairy Spoilage Bacteria and Their Lipases: Prevalence Patterns and Molecular Traits

**DOI:** 10.3390/foods15152750

**Published:** 2026-08-05

**Authors:** Yuying Lu, Qian Wu, Jinling Wang, Pradeep K. Malakar, Xu Wang, Dongmei Wang

**Affiliations:** 1 College of Food Science and Technology, Shanghai Ocean University, Shanghai 201306, China; 19816295951@163.com (Y.L.); 13540641547@163.com (Q.W.); pkmalakar@shou.edu.cn (P.K.M.); 2 International Research Center for Food and Health, Shanghai Ocean University, Shanghai 201306, China; 3 National Center of Technology Innovation for Dairy, Hohhot 010110, China; nfwjl@yili.com; 4 Inner Mongolia Yili Industrial Group Co., Ltd., Hohhot 010110, China

**Keywords:** dairy spoilage, meta-analysis, *Pseudomonas*, *Bacillus*, lipase, α/β hydrolase

## Abstract

Dairy product spoilage is primarily caused by microbial contamination and lipase activity. Systematically assessing contamination risks and elucidating the structural and functional characteristics of key lipases form a crucial foundation for achieving precise prevention and control. This article integrated 79 studies (2003–2025) to preliminarily explore the geographical distribution characteristics of spoilage bacterial contamination. The combined contamination rates were relatively high in South America (89.5%), North America (88.3%) and Asia (87.3%), followed by Europe (86.0%) and Oceania (82.2%), while Africa showed a lower rate (57.6%) based on limited data. Product-level risk differences were also identified. Considering data robustness, in addition to raw milk (92.6%), milk powder also showed a high contamination rate (86.8%). The spoilage bacterial community was highly concentrated, with *Bacillus* (40.7%) and *Pseudomonas* (20.3%) as the dominant genera, and *Pseudomonas fluorescens* (21.1%) and *Bacillus licheniformis* (20.1%) as the core spoilage species. Furthermore, structural analysis of 37 key lipases demonstrated that these enzymes universally possess a hydrophobic surface, high lipid affinity, and a conserved α/β hydrolase folding conformation. Based on these predictions, we propose a structural hypothesis: these characteristics may be related to their retained activity after thermal processing. Unlike traditional meta-analyses that merely report pollution rates, and unlike independent bioinformatics studies that analyze lipases from arbitrary strains, this study ensures that the lipases analyzed have epidemiological relevance. This work provides scientific grounds for monitoring, early warning, and risk assessment of dairy spoilage. It also lays a theoretical foundation for developing novel inhibitors targeting thermostable lipases.

## 1. Introduction

Dairy products are rich in protein, fat, carbohydrates, various minerals, and vitamins. They are globally recognized as an important source of nutrition and play a key role in promoting human growth and development, maintaining bone health, and supporting basal metabolism [1]. With global economic development and heightened public health awareness, dairy consumption continues to rise, reaching approximately 840 million tonnes worldwide in 2022 [2,3]. However, rich nutritional content and favourable water activity of dairy products also render them an ideal substrate for microbial growth and enzymatic reactions. This makes them highly susceptible to spoilage during processing, storage, and distribution, leading to significant economic losses and food safety risks [4]. According to statistics, in the United States alone, economic losses from microbial spoilage of dairy products at the retail and consumer levels reach approximately $165.6 billion, representing about 17% of total food sector losses [5]. Furthermore, food safety incidents triggered by spoilage microorganisms, such as the 2010 “blue cheese” outbreak in Italy, inflict long-term damage upon industry reputation and consumer confidence [6].

Spoilage microorganisms are the primary biological factors responsible for the deterioration of dairy product quality [7]. Among these, spoilage bacteria are particularly significant in terms of both contamination rates and severity of harm. These bacteria and the enzymes they secrete (such as proteases and lipases) can directly cause deterioration in the color, odor and texture of dairy products [8]. For instance, *Pseudomonas fluorescens*, a typical psychrophile, can grow abundantly and secrete lipases even at refrigeration temperatures (4–7 °C) [9]. Lipases, also known as glycerol ester hydrolases, catalyse the hydrolysis of triacylglycerols, thereby promoting the release of free fatty acids and glycerol [10]. Such enzymes retain its activity after pasteurization or even ultra-high temperature sterilization (UHT). It continuously catalyzes the hydrolysis of milk fat during storage, releasing short-chain fatty acids such as acetic acid and caproic acid. Although acetic acid is an important source of yogurt flavor, caproic acid can cause irreversible sensory deterioration such as acidification and off-odors during storage, leading to the “post-sterilization spoilage” phenomenon of dairy products, which severely restricts the shelf life and commercial value of dairy products [11]. Therefore, systematically understanding the contamination patterns of dairy spoilage bacteria and elucidating the molecular characteristics of their lipases at the molecular level form the foundation for achieving precise prevention and control of dairy spoilage.

Although numerous studies have investigated the contamination of spoilage bacteria in dairy products from various perspectives, these investigations exhibit certain limitations in both scope and depth. From the perspective of research, the existing works mostly focus on a single bacterial strain or product type. For instance, Marcela et al. focused on the *Pseudomonas* genus in Brazilian and Chinese milk [12]; Roberto et al. investigated the *Bacillus cereus* in the cheese processing environment [13]. Such isolated studies have resulted in highly fragmented knowledge in this field. Currently, there is a lack of meta-analyses covering the prevalence patterns and dominant bacterial communities of spoilage bacteria in dairy products from multiple regions and different categories. In terms of research depth, most studies remain confined to phenotypic descriptions such as strains identification and contamination rate statistics [8,14], with a notable absence of systematic investigations into the molecular characteristics of lipases produced by these key spoilage bacteria. This knowledge gap at the molecular mechanism level severely constrains the development of precise prevention and control strategies based on enzyme structure and function.

Therefore, this study follows a two-step progressive approach. Firstly, we employed a meta-analysis to integrate research on spoilage bacterial contamination in dairy products from 2003 to 2025. This assessed the overall prevalence of spoilage bacteria in dairy products, revealed geographical distribution and product type variations, and identified the predominant spoilage bacterial strains present. Building directly on these meta-analysis results, we then conducted systematic bioinformatics analysis specifically on the lipases secreted by the top eleven identified spoilage bacteria. This design ensures that lipase we analysis are epidemiologically relevant rather than arbitrarily selected. Our analysis encompassed conserved domains, physicochemical properties, and three-dimensional structures, aiming to elucidate the molecular-level structural and functional commonalities of these lipases. By establishing this causal chain from contamination patterns to molecular traits, this research provides a reliable theoretical foundation and a solid data basis for monitoring and controlling spoilage risks in dairy products.

## 2. Materials and Methods

### 2.1. Search Strategy and Literature Screening Process

**Figure 1:**
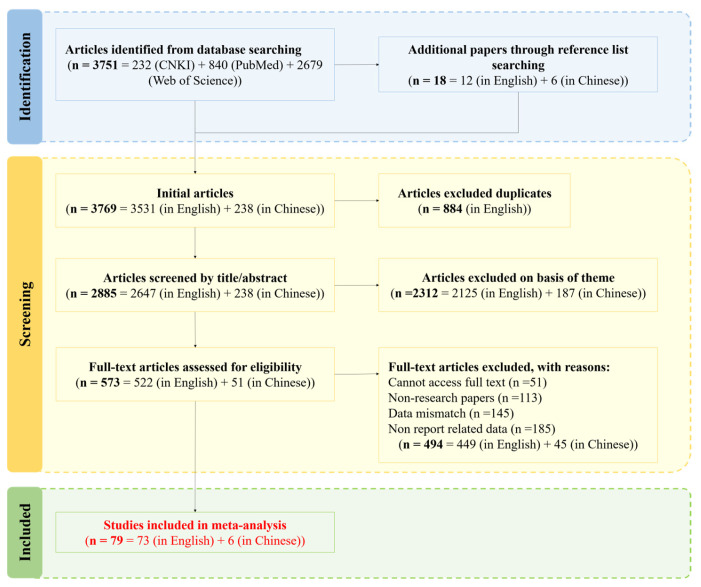
Flow diagram according to the PRISMA method.

This study strictly adheres to the standard systematic process established by the PRISMA (Preferred Reporting Items for Systematic Reviews and Meta-Analyses) guidelines (http://www.prisma-statement.org/ for details (accessed on 2 May 2026)) [15]. The study followed the operational steps outlined in Figure 1: (a) database searching and categorization of potentially relevant articles; (b) assessment of article relevance; (c) quality evaluation; (d) data extraction. This paper systematically screened article concerning the contamination of dairy products by spoilage bacteria. The databases searched included PubMed, Web of Science, and China National Knowledge Infrastructure (CNKI), covering the time period from 1 January 2003 to 31 December 2025. Chinese search terms include “spoilage bacteria”, “dairy products”, “milk”, “yogurt”, “cheese”, “butter”, “milk powder”, “cream”, “condensed milk”, “ice cream”, and “milk beverages”; English search terms include “spoilage bacteria”, “dairy products”, “milk”, “cheese”, and “yogurt”. For detailed search strategies, please refer to Table A1.

The initial search yielded a total of 3751 articles, including 232 from the CNKI, 840 from the PubMed, and 2679 from the Web of Science. An additional 18 articles were obtained by searching reference lists. After deduplication, 2885 articles remained. Based on title and abstract screening, 2312 articles were excluded as irrelevant to the study theme. Full-text retrieval and re-screening were conducted on the remaining 573 articles. Inclusion criteria were: (1) studies investigating the prevalence of spoilage bacteria in dairy products; (2) provision of extractable sample sizes and positive detection data; (3) original research articles (excluding reviews, conference abstracts, etc.). Ultimately, 79 articles were included for subsequent analysis.

### 2.2. Data Extraction and Quality Assessment

The following information was extracted from each included article: title, authors, country/region of study, year of publication, type of dairy product, sample size, number of positive samples for spoilage bacteria, spoilage bacterial strains, and detection methods. The Newcastle-Ottawa Scale (NOS) (https://www.ohri.ca/programs/clinical_epidemiology/oxford.asp (accessed on 2 May 2026)) was used to assess the quality of the included articles. The scale evaluates studies across three dimensions (sample selection, comparability, and outcome assessment) and consists of nine items (Table A2). Based on the number of items, the article was classified into three quality levels: studies meeting ≥ 7 items were considered high quality, those meeting 4–6 items were of moderate quality, and those meeting ≤3 items were classified as low quality.

### 2.3. Data Analysis

Adhering to the fundamental principles and standardized procedures of meta-analysis, this study developed a systematic data extraction form to collect information from the included articles. All statistical analyses were performed using Microsoft Excel. In this study, Cochran’s Q test, the I^2^ statistic, and 95% confidence intervals were used to assess heterogeneity. Generally, *p* ≥ 0.1 in Cochran’s Q test indicates no heterogeneity, while *p* < 0.1 indicates the presence of heterogeneity among studies. The I^2^ statistic measures the percentage of total variation in effect sizes that is attributable to true heterogeneity between studies (rather than chance factors). Conventionally, I^2^ values of 25%, 50%, and 75% are used as thresholds for low, moderate, and high heterogeneity, respectively. An I^2^ greater than 50% is considered to indicate substantial heterogeneity, and an I^2^ greater than 75% is considered to indicate considerable heterogeneity. Since this is a meta-analysis of single-group rates with no control group and therefore no positive or negative outcomes, the results are largely descriptive. Accordingly, a random-effects model was employed for all meta-analyses [16]. Comprehensive Meta-Analysis 3.0 (CMA 3.0) software was employed to calculate the pooled effect size, 95% confidence interval, *p* value, and Q value.

### 2.4. Publication Bias Test

Publication bias refers to the phenomenon where research results with statistical significance are more likely to be reported and published compared to those with non-significant or null findings. It constitutes one of the primary factors affecting the validity of meta-analysis outcomes [17]. In meta-analyses, funnel plots are frequently employed to visually assess publication bias. However, funnel plot asymmetry is not solely attributable to publication bias; heterogeneity is also recognized as a significant factor contributing to such asymmetry. When studies exhibit high heterogeneity (I^2^ > 75%), relying solely on funnel plots to assess the applicability of publication bias is of limited value. Therefore, to mitigate the subjectivity inherent in graphical interpretation, this study employs Fail-safe N analysis (N*_fs_*) [18] combined with Egger’s regression test [19] for publication bias testing, thereby enhancing the objectivity and accuracy of the results.

(1) Fail-safe N analysis

Fail-safe N analysis aims to assess the number of unpublished studies required to nullify the statistical significance of the current meta-analysis results, thereby evaluating the robustness of the findings. The N*_fs_* values are calculated at significance levels of α = 0.05 and α = 0.01, using the following formula:

(1) $${N_{fs}}_{0.05}={\left(\sum \frac{z}{1.64}\right)}^{2}-K$$

(2) $${N_{fs}}_{0.01}={\left(\sum \frac{z}{2.33}\right)}^{2}-K$$

Among these, *z* denotes the z-score for each individual study and *K* represents the number of included articles. Should N*_fs_* exceed the critical threshold (5K + 10), the meta-analysis findings are considered more reliable with reduced publication bias influence; conversely, insufficient stability indicates potential bias risks in the conclusions.

(2) Egger’s regression test

Egger’s regression test identifies publication bias by examining the symmetry of funnel plots. This method establishes a linear regression model with precision as the independent variable and Standard Normal Deviate (SND) as the dependent variable:

(3) SND=A+B×Precision

Among these, *A* denotes the intercept, reflecting the asymmetry of the funnel plot; *B* represents the slope, indicating the magnitude of the effect size. Should the *p* value for the intercept term in the regression equation be less than 0.05, this indicates the presence of significant publication bias; conversely, a higher *p* value suggests a lower risk of bias. Both tests may be conducted using the Comprehensive Meta-Analysis statistical software.

### 2.5. Screening and Physicochemical Characterization of Lipase from Spoilage Bacteria

To obtain the sequences and physicochemical information of lipases from predominant spoilage bacteria in dairy products, we first searched the NCBI Protein Database using the names of predominant spoilage bacterial strains (identified through meta-analysis) in combination with the keyword “Lipase” to retrieve amino acid sequences. Subsequently, the ProtParam within the ExPASy online platform (http://web.expasy.org/protparam/ (accessed on 2 May 2026)) was employed to analyze the physicochemical properties of the obtained lipase sequences. This yielded key parameters including relative molecular weight, theoretical isoelectric point (pI), aliphatic index, and average hydrophilicity (GRAVY), thereby establishing a data foundation for subsequent structural and functional investigations.

### 2.6. Domain and Functional Analysis of Lipase from Spoilage Bacteria

Based on the lipase amino acid sequence obtained from NCBI, three-dimensional structure prediction was performed using the SWISS-MODEL online homology modelling platform (https://swissmodel.expasy.org/ (accessed on 2 May 2026)). This platform automatically aligns the target sequence against a database of known protein structures, selecting the template with the highest similarity to construct a three-dimensional structural model of lipase. To facilitate subsequent structural analysis, the resulting model was visualized using the PyMOL 3.1 molecular graphics software.

## 3. Results

### 3.1. Characteristics and Quality Assessment of Included Studies

A total of 79 articles that were ultimately included in the analysis (Table A3) are summarized in terms of their basic characteristics in Table 1. These articles spanned 27 countries across six continents. Based on the processing technology and product form, the dairy products included in this study were classified into five categories: (1) Liquid milk, including raw milk, pasteurized milk, ultra-high temperature milk (UHT milk), refrigerated milk, extended shelf life milk (ESL milk), bactofuged milk, and yogurt; (2) Milk powder, including milk powder and dried colostrum; (3) Milk fat products, including cream and butter; (4) Ice cream; and (5) Cheeses. According to the assessment using the Newcastle-Ottawa Scale (NOS) (Table A2), the overall methodological quality of the included studies was relatively high, with over 90% of the studies receiving good scores in terms of sample selection and quality reporting. However, 6.33% of the studies had deficiencies in reporting sample size, suggesting that future research should further improve the completeness and transparency of methodological descriptions.

### 3.2. Geographic Distribution Characteristics of Spoilage Bacteria in Dairy Products

**Figure 2:**
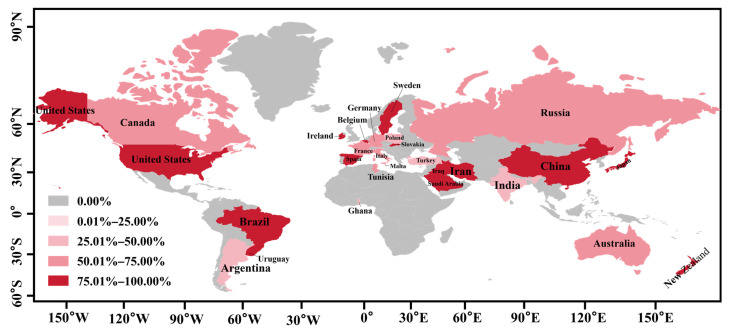
Geographical distribution of spoilage bacteria in dairy products.

The meta-analysis results showed significant regional heterogeneity in the contamination rate of spoilage bacteria in dairy products (Figure 2, Table 2). The highest contamination level was found in South America (89.5%), followed by North America (88.3%), Asia (87.3%), Europe (86.0%) and Oceania (82.2%), while Africa had a relatively lower contamination rate (57.6%).

Fifteen studies from South America involved Argentina, Brazil, and Uruguay. The overall prevalence in this region was 89.5% (95% CI: 0.78–0.96), with high heterogeneity (I^2^ = 85.20%). Brazil contributed the largest number of studies (12 studies), with a detection rate of 94.9% (95% CI: 0.82–0.99), making it the main contributor to the South American data. The detection rates for Argentina and Uruguay differed significantly, at 39.9% (95% CI: 0.20–0.64) and 83.3% (95% CI: 0.19–0.99), respectively. However, due to the limited number of studies included from these two countries, their statistical precision is limited, and the results should be interpreted with caution.

Among the 12 studies included from North America, the vast majority originated from the United States, with a pooled detection rate of 89.1% (95% CI: 0.71–0.97). The extremely high heterogeneity (I^2^ = 98.80%) suggests substantial variation in contamination levels across different cities or dairy product types within the United States. Only one study from Canada reported a detection rate of 75.0%. Given that the studies were predominantly from the United States, the representativeness of the North American data is limited.

A total of six studies were included from Oceania (four from Australia and two from New Zealand), with a pooled detection rate of 82.2% (95% CI: 0.60–0.94) but extremely high heterogeneity (I^2^ = 93.33%). New Zealand had a higher detection rate than Australia, with lower heterogeneity (I^2^ = 68.11%). However, since all studies from Oceania came only from Australia and New Zealand, lacking data from other countries, this pooled detection rate has limited representativeness for Oceania as a whole.

A total of 18 studies were included from Asia, with an overall prevalence of 87.3% (95% CI: 0.72–0.95) and high heterogeneity (I^2^ = 87.72%). Among these, Iran had the highest contamination rate at 99.5% (95% CI: 0.93–1.00), while India had the lowest at 35.1% (95% CI: 0.25–0.47). Of note, the Asian data were mainly concentrated in China, thus having limited representativeness for other regions.

The overall prevalence of spoilage bacteria in European dairy products was 86.0% (95% CI: 0.75–0.94), with Italy contributing the largest number of studies (six studies). Most countries showed relatively high detection rates, such as Malta (90.0%, 95% CI: 0.33–0.99), Belgium (98.6%, 95% CI: 0.91–1.00), and Spain (97.8%, 95% CI: 0.86–1.00). Notably, the detection rates in Poland and Turkey were significantly lower than those in other European countries, at only 34.0% (95% CI: 0.23–0.47) and 28.8% (95% CI: 0.21–0.38), respectively, indicating substantial regional differences in contamination levels within Europe.

A total of four studies were included from Africa, with a pooled detection rate of 57.6% (95% CI: 0.30–0.81). Research in this region was mainly concentrated in Tunisia (three studies), and heterogeneity was high (I^2^ = 91.46%).

The following limitations should be noted when interpreting the above results. First, the number of included studies from some continents is limited (e.g., only four from Africa and six from Oceania), resulting in wide confidence intervals for the relevant estimates and insufficient statistical precision. Second, the geographical distribution of studies is highly concentrated: Most North American studies come from the United States, 80% of South American studies came from Brazil, 75% of African studies came from Tunisia, Oceania studies came only from Australia and New Zealand, and Asian studies were mainly from China. This data concentration means that the pooled estimates have very limited representativeness for their respective continents as a whole. Third, heterogeneity across studies is generally high (I^2^ > 75%), suggesting that contamination levels may be strongly influenced by factors such as local climate, production conditions, and detection systems. In summary, the intercontinental comparisons presented in this study should be regarded as exploratory findings rather than definitive conclusions regarding contamination levels across continents. Future monitoring studies are needed in more geographical regions to validate these findings.

### 3.3. Differences in Contamination Risks Among Various Dairy Product Types

Significant variations in spoilage bacteria contamination rates were observed across different dairy product types (Table 3). Among liquid milk, raw milk showed the highest contamination rate at 92.6%. It should be noted that raw milk naturally contains indigenous milk lipases, whose activity can also contribute to dairy spoilage. Therefore, the contamination rate for raw milk serves only as a reference for microbial contamination levels and should not be directly compared with other dairy products. Among other liquid milk products, ESL milk had a contamination rate of 87.5%, refrigerated milk 83.9%, yogurt 71.1%, and pasteurized milk 57.5%. UHT milk and bactofuged milk showed relatively low contamination rates (28.9% and 10.0%, respectively). In addition, one study reported a contamination rate of 90.0% for fluid milk without specifying the type. The combined contamination rate of milk powder is 86.8%, while that of dried colostrum is 93.8%. In milk fat products, the contamination rates of cream and butter are 95.8% and 20.0% respectively, but the data volume is very small and should be regarded as preliminary results. The contamination rate of ice cream is relatively low (29.2%); the contamination rate of cheese is at a medium level (46.5%). However, only a few study types (such as ESL milk, refrigerated milk, bactofuged milk, butter, ice cream, dried colostrum) with statistical values should be regarded as preliminary results and need to be verified by future studies with larger sample sizes. Furthermore, included studies rarely reported the fat content of the tested dairy products (e.g., full-fat, semi-skimmed, skimmed). Given that fat content is an important factor influencing lipolytic spoilage, its impact on contamination rates could not be analyzed due to the limitations of the original data. Overall, high-moisture, non-frozen products (e.g., liquid milk) are more likely to serve as vehicles for microbial proliferation, while processing and storage conditions (e.g., drying, freezing) can significantly influence spoilage levels. This reflects that different dairy products exhibit distinct patterns of microbial contamination risk due to variations in raw material characteristics, processing techniques, and storage conditions [20,21]. Furthermore, the microbial standards in the EU Regulation EC 2073/2005 mainly set limits for hygiene indicator bacteria (such as Enterobacteriaceae) and pathogenic microorganisms (such as Coagulase-positive staphylococci) in products like pasteurized milk, milk powder, ice cream, and cheeses [22]. This study, however, focuses on the contamination levels of spoilage bacteria. Therefore, it is possible to consider a comprehensive assessment of the microbial quality of dairy products in the future (taking into account both pathogenic and spoilage bacteria).

### 3.4. Analysis of Primary Spoilage Bacterial Genera and Strains in Dairy Products

**Figure 3:**
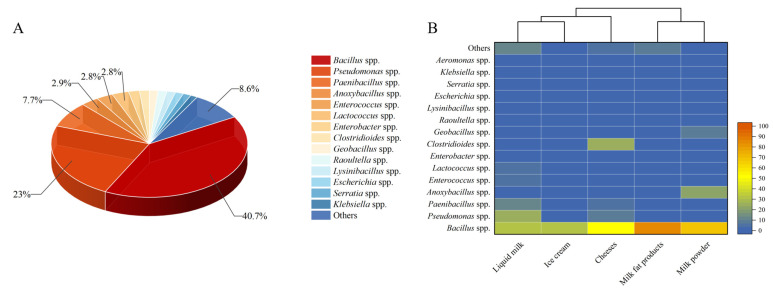
Composition and distribution of spoilage bacterial genera in dairy products. (**A**) Genus-level composition of spoilage bacteria in dairy products. (**B**) Clustering heatmap of spoilage bacterial genera in different dairy product categories.

This study identified 77 genera of spoilage bacteria through statistical analysis. As shown in Figure 3A, the distribution of spoilage bacteria in dairy products exhibits a highly concentrated pattern. *Bacillus*, accounting for 40.7%, emerged as the predominant spoilage bacterial group, followed by *Pseudomonas* at 23.0%. Together, these two genera accounted for over 60% of the total, constituting the dominant bacterial community responsible for dairy product spoilage. Other genera such as *Paenibacillus* (7.7%), *Anoxybacillus* (2.9%), and *Enterococcus* (2.8%) were detected but each accounted for less than 10.0%. Notably, among non-dominant genera, 62 genera exhibited very low proportions. Consequently, this study grouped them under the broad category of “others”, which accounted for 8.6% of the total. Figure 3B further reveals the core status of *Bacillus*. In addition, *Pseudomonas* and *Paenibacillus* showed relatively high abundance in liquid milk, *Clostridioides* were more prominent in cheeses, and *Anoxybacillus* accounted significantly in milk powder. These findings indicate that product characteristics significantly influence the composition of spoilage bacterial communities.

This study conducted a comprehensive analysis of spoilage bacteria strains reported in 79 literature sources, encompassing a total of 290 bacterial strains (Table A4). To enhance the representativeness of findings, analysis focused on spoilage bacteria strains documented in more than five studies. The detection rate data for bacterial strains in Table 4 further clarifies the contamination characteristics of dairy products. The combined detection rates for *Pseudomonas fluorescens* and *Bacillus licheniformis* were highest at 21.1% (95% CI: 0.15–0.29) and 20.1% (95% CI: 0.16–0.25) respectively, making them the most prevalent spoilage bacteria in dairy products. Both are closely associated with dairy product deterioration (e.g., proteolysis, fat oxidation), and their high heterogeneity (*p* < 0.05, I^2^ > 90%) suggests contamination levels may be influenced by geographical location, product type, or detection methods. *Bacillus cereus* (19.4%, 95% CI: 0.14–0.26) and *Bacillus subtilis* (11.7%, 95% CI: 0.08–0.17) were also prevalent, further highlighting the predominance of the genus *Bacillus* in dairy spoilage.

Additionally, within the genus *Pseudomonas*, *Pseudomonas fragi* (10.2%, 95% CI: 0.07–0.15), *Pseudomonas putida* (8.3%, 95% CI: 0.04–0.15), and *Pseudomonas lundensis* (7.5%, 95% CI: 0.03–0.17) also exhibited detectable rates, reflecting the genus’s broad adaptability in dairy processing and storage stages. *Lactococcus lactis* (10.5%, 95% CI: 0.05–0.23), *Enterococcus faecalis* (6.5%, 95% CI: 0.11–0.30), and *Enterococcus faecium* (7.0%, 95% CI: 0.05–0.10) exhibited relatively lower detection rates. However, their confirmed spoilage potential (e.g., gas production and acid spoilage) warrants attention in Yoghurt and cheese.

**Figure 4:**
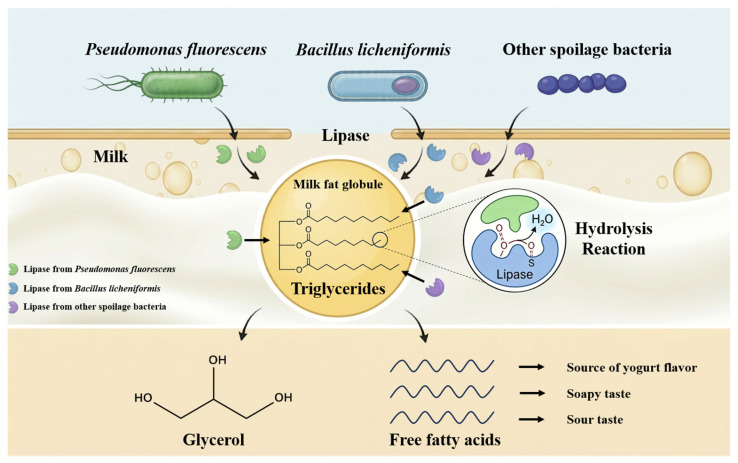
Biochemical mechanisms of dairy spoilage caused by bacterial lipases.

In summary, the eleven predominant spoilage bacteria identified in dairy products in this study are: *Pseudomonas fluorescens*, *Bacillus licheniformis*, *Bacillus cereus*, *Bacillus subtilis*, *Lactococcus lactis*, *Pseudomonas fragi*, *Pseudomonas putida*, *Bacillus pumilus*, *Pseudomonas lundensis, Enterococcus faecium* and *Enterococcus faecalis*. To illustrate the biochemical mechanisms by which these predominant spoilage bacteria cause dairy spoilage, a schematic diagram is presented in Figure 4.

To assess the extent to which the meta-analysis results of this study were influenced by publication bias, tests were conducted for each analysis group, and the results are shown in Table A5. Fail-safe N analysis indicated that all groups had values far exceeding the critical thresholds, suggesting that the overall results were minimally affected by publication bias and exhibited high stability. Egger’s regression test revealed potential publication bias in four groups related to the conclusions (the prevalence of *Lactococcus lactis*, *Pseudomonas putida*, *Pseudomonas lundensis*, and *Enterococcus faecalis*). However, Fail-safe N for these four groups were all far above the critical values, indicating that even in the presence of publication bias, the current conclusions remain highly robust. Considering the results of both methods together, the influence of publication bias on the main conclusions of this study is generally manageable, and most of the analytical results demonstrate good stability.

Based on the above analysis, we observed consistently high I^2^ values (>75%) in most subgroups. In a large-scale meta-analysis covering 27 countries, spanning over twenty-two years and with diverse study designs, this level of high heterogeneity was in line with expectations. The high heterogeneity may stem from: geographical and climatic differences; variations in dairy processing techniques; differences in detection methods; and variations in study quality and sample size. More importantly, the random-effects model used in this study provides more conservative pooled estimates (with wider confidence intervals). Additionally, the higher safety factor also indicates that, despite the heterogeneity, our pooled estimates remain robust.

### 3.5. Analysis of the Conserved Domains of Lipase Produced by Spoilage Bacteria in Dairy Products

Based on the aforementioned findings, this study identified 11 predominant spoilage bacterial strains. By cross-referencing with the NCBI database, 37 lipase sequences associated with these spoilage bacteria were further screened (Table A6). Analysis of the conserved domains within the screened lipase sequences revealed a total of 18 distinct conserved domains. Twenty-eight lipases contained only one conserved domain, eight lipases contained two conserved domains, and only one lipase contained three conserved domains. Among these, lipases containing the Abhydrolase superfamily conserved domain were the most prevalent (11 items). This domain belongs to the α/β hydrolase fold superfamily and is a potential regulator of lipid metabolism and signal transduction [23]. The second most prevalent was the CVT17 superfamily (6 items), a domain playing a pivotal role in autophagosome-lysosome degradation [24], primarily involved in intracellular transport and vesicular secretion.

Five lipases contain the conserved SGNH_hydrolase superfamily domain. Enzymes within this family possess flexible active sites capable of undergoing conformational changes upon substrate binding [25]. Four lipases contain the Aes superfamily conserved domain, while three possess the EstA superfamily conserved domain. The EstA superfamily domain exhibits specificity for short-chain acyl substrates [26], and both domains participate in lipid transport and metabolism. Lipases containing conserved domains of the DAP2 superfamily, Lip2 superfamily, or MenH superfamily (two from each) are primarily involved in the transport and metabolism of amino acids, lipids, and coenzymes, respectively.

Additionally, lipases containing the CusA superfamily, FrmB superfamily, KxYKxGKxW_sig superfamily, HemolysinCabind superfamily, PldB superfamily, COG2931 superfamily, COG4782 superfamily, Axe1 superfamily, CwlO1 superfamily, and CHAP superfamily were the least abundant (one each). The CusA superfamily comprises Cu and Ag efflux pumps. It is primarily involved in inorganic ion transport and metabolism. The FrmB superfamily has a defense function. The PldB superfamily shares the same function as the EstA superfamily and Lip2 superfamily. The COG2931 superfamily consists of calcium-binding proteins associated with RTX toxins. Its function is similar to that of the Axe1 superfamily, primarily participating in the synthesis, transport, and degradation of secondary metabolites. The CHAP superfamily exhibits amidase-like activity and is involved in bacterial cell wall metabolism. The functions of the KxYKxGKxW_sig superfamily, HemolysinCabind superfamily, CwlO1 superfamily, and COG4782 superfamily remain uncharacterized.

### 3.6. Analysis of the Physicochemical Properties of Lipase Produced by Spoilage Bacteria in Dairy Products

According to Table A6, the physicochemical properties of the lipases showed the following characteristics: The molecular weight distribution was broad, ranging from 24.12 to 86.82 kDa. The amino acid sequence lengths varied significantly, from 212 to 776 a.a. The isoelectric points also showed a wide range, from 4.10 to 9.68. Most of the lipases had acidic or neutral isoelectric points. The average hydrophilicity of all 36 lipases is negative, indicating a hydrophobic surface characteristic. This structural feature facilitates interaction with lipid substrates. Lipophilic coefficients are generally high (69.11–100.87), further confirming the strong affinity of this enzyme class for lipid matrices. Furthermore, lipases exhibited marked variations in instability coefficients, a property potentially impacting their tolerance and stability within food processing environments.

### 3.7. Structural Prediction Analysis of Lipase Produced by Spoilage Bacteria in Dairy Products

**Figure 5:**
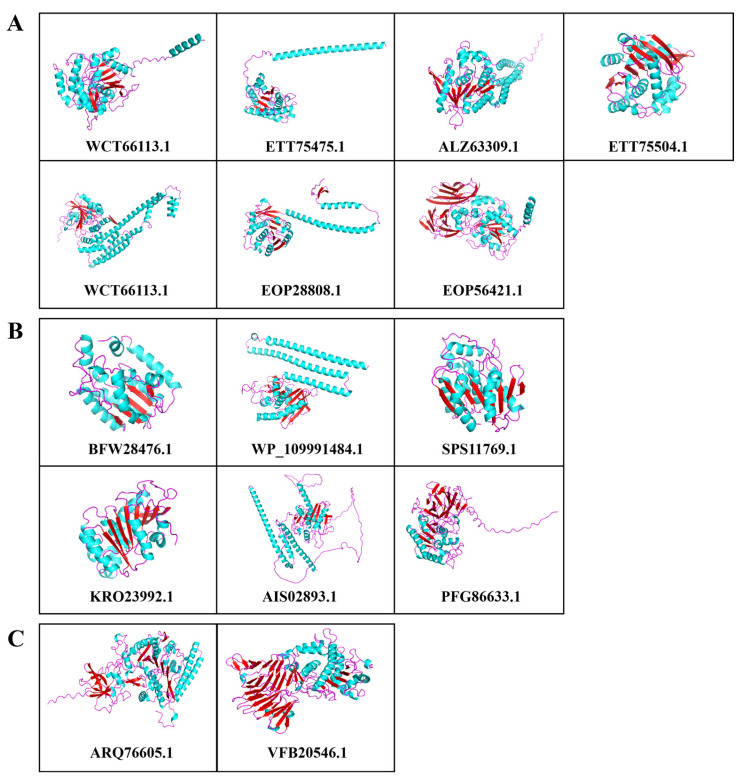
Three-dimensional structural models of lipases from *Bacillus cereus*, *Lactococcus lactis* and *Pseudomonas fragi*. (**A**) Homology model of lipase from *Bacillus cereus*. (**B**) Homology model of lipase from *Lactococcus lactis*. (**C**) Homology model of lipase from *Pseudomonas fragi*. The secondary structure elements are color-coded: cyan for α-helices, red for β-sheets, and purple for loops.

**Figure A1:**
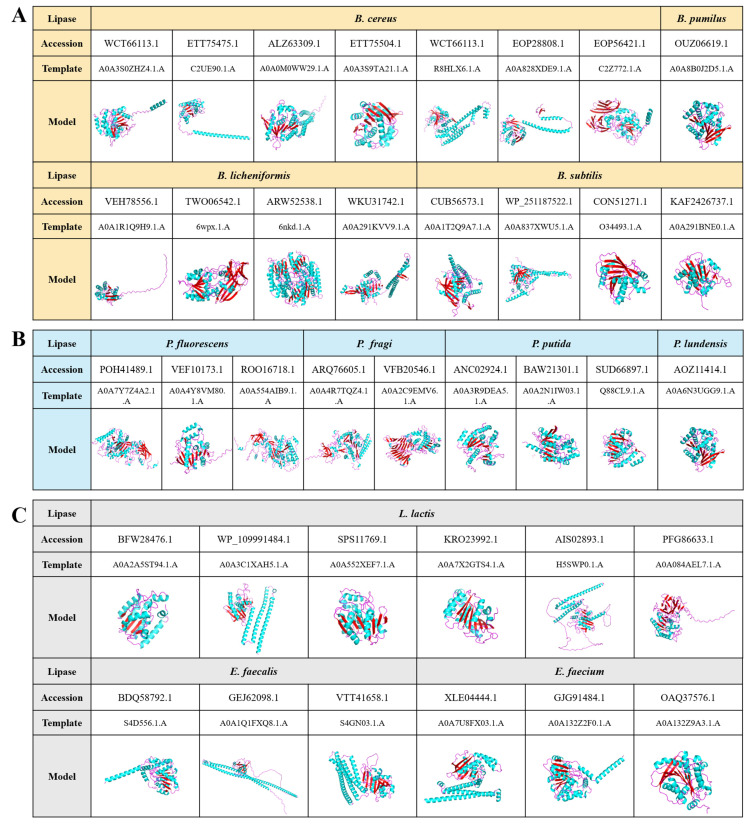
Three-dimensional structural models of lipases from major dairy spoilage bacteria. (**A**) Homology model of lipase from *Bacillus*. (**B**) Homology model of lipase from *Pseudomonas*. (**C**) Homology model of lipases from other bacteria. The secondary structure elements are color-coded: cyan for α-helices, red for β-sheets, and purple for loops.

This study employed the Swiss-Model online tool to perform homology modelling on the aforementioned 37 lipase sequences, with visualization completed using PyMOL (Figure A1). The homology modelling results revealed that each of the 37 lipases matched distinct templates. All lipases exhibited a characteristic α/β-folded structure, wherein β-sheets formed a central backbone surrounded by multiple α-helices. These β-sheets predominantly adopted a parallel arrangement, maintaining consistent orientation and high degree of twist. Lipases from different sources exhibit variations in the number and positioning of α-helices, as well as the quantity of β-strands, thereby conferring distinct catalytic properties [9,27]. For instance, the lipase from *Pseudomonas fragi* (GenBank: VFB20546.1) possesses 32 β-strands and 19 α-helices; conversely, the lipase from *Bacillus cereus* (GenBank: ETT75504.1) possesses only 10 β-sheets and 11 α-helices. Within the three-dimensional models of lipases, the N-terminal structures exhibit diversity, comprising either helical or ring-like conformations. We observed that the lipases from *Pseudomonas cereus* (except GenBank: ETT75504.1) and *Lactococcus lactis*, which exhibit lower instability coefficients, both possess extended ring-like conformations at their N-termini (Figure 5). The maintenance of overall conformation depends on the integrity of the hydrophobic core and the hydrogen bond network. However, the flexibility of the N-terminus may affect protein stability to some extent. Future studies could consider truncating or modifying this region to verify its specific contribution to stability. It should be noted that the above structural observation results are derived from homology modeling and predictions, rather than experimental determination. The identified α/β hydrolase fold and hydrophobic surface features provide a reasonable structural explanation for thermal stability. However, these remain hypotheses and do not constitute experimental evidence. Future experimental studies are needed to validate this hypothesis.

## 4. Discussion

Through an integrated approach combining meta-analysis with bioinformatics, this study systematically characterizes the prevalence patterns of spoilage bacteria in dairy products and the shared molecular features of their key lipases. Findings indicate that contamination by spoilage bacteria in dairy products exhibits significant geographical and product-specific heterogeneity, with spoilage bacterial communities predominantly concentrated within the genera *Bacillus* and *Pseudomonas*. Further analysis indicates that the lipases secreted by these dominant strains possess conserved hydrophobic interfaces and stable three-dimensional folding structures. This series of discoveries, spanning from macro-level prevalence patterns to micro-level molecular characteristics, provides new perspectives and evidence for deepening our understanding of dairy product spoilage mechanisms and developing precise prevention and control strategies.

First of all, the contamination and distribution of spoilage bacteria in dairy products exhibit significant geographical and product heterogeneity. Meta-analysis results showed that the pooled detection rates were higher in South America (89.5%), North America (88.3%), and Asia (87.3%), while the pooled detection rate in Africa was lower (57.6%). However, the interpretation of these geographical differences is subject to limitations. First, the number of included studies and the geographical coverage across continents are unbalanced: Almost all North American studies came from the United States; twelve of the fifteen South American studies came from Brazil; the six Oceania studies came only from Australia and New Zealand; and three of the four African studies came from Tunisia. Therefore, these pooled detection rates essentially reflect the situation in these few countries and have limited representativeness for their respective continents as a whole. Second, high heterogeneity is commonly observed across studies from different countries, suggesting that regional climate, production practices, and detection systems may jointly influence contamination levels [28]. However, due to the limitations of the available data, the specific roles of these influencing factors require further investigation. In summary, the geographical differences presented in this study should be regarded as exploratory findings rather than definitive conclusions regarding contamination levels across continents.

Secondly, from the perspective of product type, the contamination rates of different processing methods of liquid milk vary significantly: with increasing heat treatment intensity, the contamination rate dropped from 57.5% in pasteurized milk to 28.9% in UHT milk. This trend indicates that the heat treatment intensity is a key factor in controlling microbial contamination in dairy products [29]. Additionally, the contamination rate of milk powder reached 86.8%, suggesting that low water activity alone cannot completely control the risk of spoilage. Other key influencing factors such as storage conditions and pH also need to be considered [30]. In conclusion, differentiated control should be implemented based on product characteristics. It should be noted that factors such as fat content of products, type of cheese (such as raw material and ripeness), and whether exogenous lipases are added during processing all affect the risk of spoilage. However, most of the article does not report this information, and it is recommended that future research should include this.

Thirdly, the structure of the spoilage bacterial community in dairy products is highly concentrated. *Bacillus* and *Pseudomonas* are the dominant genera, together accounting for more than 60% of the total. This finding corroborates multiple regional studies and review conclusions in recent years [4], reflecting the broad adaptability and ecological competitive advantage of these two bacterial groups within the dairy product environment. Among these, *Pseudomonas fluorescens* (21.1%) and *Bacillus licheniformis* (20.1%) emerged as the predominant strains, indicating their pivotal role in the spoilage process. From a physiological perspective, *Pseudomonas*, as a typical psychrophile, maintains metabolic activity under refrigerated conditions and secretes thermostable lipases and proteases. This leads to acid spoilage in milk during low-temperature storage [31]. Relying on their heat-resistant spores, *Bacillus* species can withstand conventional thermal processing and persist in products. They thus become major spoilage factors in low-moisture products such as milk powder [32]. This finding provides a clear target for targeted monitoring and sterilization process optimization. It is essential to evaluate the efficacy of existing thermal sterilization protocols in inactivating spores and to strengthen the entire cold chain to inhibit the proliferation of psychrophilic bacteria.

More importantly, this study identified the common structural characteristics of lipases from predominant spoilage bacteria at the molecular level. Based on these computational predictions, we propose a hypothesis: these lipases generally possess hydrophobic surfaces, high lipid affinity, and a conserved α/β hydrolase folding conformation, which may be related to their thermal stability. Three-dimensional modeling revealed that the Abhydrolase superfamily lipases exhibit a typical α/β hydrolase folding conformation, with a “lid” structure composed of α-helices present above the catalytic triad [33]. We speculate that this structure may affect the enzyme’s interface activation ability and thermal stability by regulating the active center. It should be emphasized that the above structure-function relationship is derived from homology modeling and prediction, not experimentally proven. Therefore, it should be regarded as a hypothesis that needs to be verified. This study expands the understanding of spoilage mechanisms from the microbial survival level to a new dimension of enzyme molecular stability. Furthermore, the lipase sequences were obtained from database reference strains, not directly sequenced from the isolates in the included studies. Future research should consider direct sequencing of lipase genes from spoilage isolates.

## 5. Conclusions

This study conducted a meta-analysis on the prevalence of spoilage bacteria in dairy products across different regions and categories. We identified the predominant spoilage bacterial strains and revealed the conserved structural features of their lipases through bioinformatics analysis. Appropriate processing methods (such as heat treatment) are indeed one of the key factors in reducing the prevalence of spoilage bacteria in dairy products: the contamination rate of pasteurized milk is significantly higher than that of UHT milk. However, relying solely on a single processing method cannot completely control spoilage; multiple factors such as water activity, storage temperature, fat content, and pH should be comprehensively controlled. The hydrophobic surface and high lipid affinity of the predominant spoilage bacterial lipases may explain their thermal stability. This finding offers a potential structural explanation for the “post-sterilization spoilage” phenomenon. Based on these findings, we suggest designing specific rapid detection techniques targeting the conserved structural domains and folding conformations of lipases, and developing targeted inhibition techniques based on conformational induction of inactivation. These data will provide important theoretical value for future research in this field and offer multi-level strategic insights for the monitoring and prevention of dairy product spoilage risks.

## Figures and Tables

**Table 1:** Characteristics of included studies.

Data Features		Number of Articles
Study region	North America	12
	Oceania	6
	Africa	4
	South America	15
	Europe	24
	Asia	18
Product type	Liquid milk	79
	Milk powder	13
	Milk fat products	4
	Cheeses	21
	Ice cream	2
Key spoilage bacteria	*Bacillus cereus*	35
	*Bacillus licheniformis*	27
	*Pseudomonas fluorescens*	30
	*Bacillus subtilis*	21
	*Bacillus pumilus*	18
	*Pseudomonas fragi*	14
	*Pseudomonas putida*	14
	*Lactococcus lactis*	13
	*Pseudomonas lundensis*	11
	*Pseudomonas aeruginosa*	8
	*Enterococcus faecalis*	8
	*Bacillus circulans*	6
	*Enterococcus faecium*	6
	*Pseudomonas gessardii*	7
	*Pseudomonas psychrophila*	6
	*Pseudomonas veronii*	6

**Table 2:** Detection rate of spoiled dairy products by region.

Continent	Country	Number of Articles	Pooled Detection Rate (95% CI)	Heterogeneity	
				*p* Value	I^2^ (%)
North America		12	88.3% (0.67–0.97)	0.00	97.82
	United States	11	89.1% (0.71–0.97)	0.00	98.80
	Canada	1	75.0% (0.59–0.86)	1.0 0	0.00
Oceania		6	82.2% (0.60–0.94)	0.00	93.33
	Australia	4	70.9% (0.43–0.89)	0.00	94.52
	New Zealand	2	98.8% (0.70–1.00)	0.08	68.11
Africa		4	57.6% (0.30–0.81)	0.00	87.55
	Ghana	1	46.7% (0.30–0.64)	1.00	0.00
	Tunisia	3	67.3% (0.26–0.93)	0.00	91.46
South America		15	89.5% (0.78–0.96)	0.00	85.20
	Argentina	2	39.9% (0.20–0.64)	0.01	77.66
	Brazil	12	94.9% (0.82–0.99)	0.00	79.18
	Uruguay	1	83.3% (0.19–0.99)	1.00	0.00
Europe		24	86.0% (0.75–0.94)	0.00	91.32
	Belgium	2	98.6% (0.91–1.00)	0.55	0.00
	France	2	98.7% (0.91–1.00)	0.57	0.00
	Germany	3	69.1% (0.40–0.88)	0.11	44.15
	Ireland	1	92.0% (0.85–0.96)	1.00	0.00
	Italy	6	66.1% (0.38–0.86)	0.00	88.16
	Malta	1	90.0% (0.33–0.99)	1.00	0.00
	Poland	1	34.0% (0.23–0.47)	0.00	80.13
	Russia	1	71.3% (0.36–0.92)	0.88	0.00
	Slovakia	1	83.3% (0.19–0.99)	1.00	0.00
	Spain	2	97.8% (0.86–1.00)	0.98	0.00
	Sweden	1	83.3% (0.69–0.92)	1.00	0.00
	Turkey	3	28.8% (0.21–0.38)	0.26	25.99
Asia		18	87.3% (0.72–0.95)	0.00	87.72
	China	13	91.7% (0.66–0.98)	0.00	82.00
	India	1	35.1% (0.25–0.47)	0.02	67.77
	Iraq	1	99.0% (0.86–1.00)	1.00	0.00
	Iran	1	99.5% (0.93–1.00)	1.00	0.00
	Japan	1	93.8% (0.46–1.00)	1.00	0.00
	Saudi Arabia	1	96.2% (0.60–1.00)	1.00	0.00

**Table 3:** Detection rates of various spoiled dairy product types.

Classification	Type	Number of Articles	Pooled Detection Rate (95% CI)	Heterogeneity	
				*p* Value	I^2^ (%)
Liquid milk	Raw milk	45	92.6% (0.87–0.96)	0.00	94.59
	Pasteurized milk	20	57.5% (0.41–0.73)	0.00	96.68
	UHT milk	4	28.9% (0.11–0.58)	0.00	90.75
	Refrigerated milk	2	83.9% (0.03–0.99)	0.00	92.05
	ESL milk	1	87.5% (0.27–0.99)	1.00	0.00
	Bactofuged milk	1	10.0% (0.05–0.18)	1.00	0.00
	Yogurt	5	71.1% (0.40–0.90)	0.09	50.73
	Type not specified	1	90.0% (0.33–0.99)	1.00	0.00
Milk powder	Milk powder	12	86.8% (0.66–0.96)	0.00	88.06
	Dried colostrum	1	93.8% (0.46–1.00)	1.00	0.00
Milk fat products	Cream	3	95.8% (0.78–0.99)	0.28	20.98
	Butter	1	20.0% (0.07–0.40)	1.00	0.00
Ice cream	Ice cream	2	29.2% (0.23–0.37)	0.51	0.00
Cheeses	Cheese	21	46.5% (0.36–0.58)	0.00	84.25

**Table 4:** Detection rates of key spoilage bacteria in dairy products.

Spoilage Strain	Number of Articles	Pooled Detection Rate (95% CI)	Heterogeneity	
			*p* Value	I^2^ (%)
*Bacillus cereus*	35	19.4% (0.15–0.28)	0.00	95.88
*Bacillus licheniformis*	27	20.1% (0.16–0.25)	0.00	94.11
*Pseudomonas fluorescens*	30	21.1% (0.15–0.29)	0.00	96.09
*Bacillus subtilis*	21	11.7% (0.08–0.17)	0.00	92.20
*Bacillus pumilus*	18	8.2% (0.05–0.13)	0.00	91.74
*Pseudomonas fragi*	14	10.2% (0.07–0.15)	0.00	83.53
*Pseudomonas putida*	14	8.3% (0.04–0.15)	0.00	93.74
*Lactococcus lactis*	13	10.5% (0.05–0.23)	0.00	94.72
*Pseudomonas lundensis*	11	7.5% (0.03–0.17)	0.00	95.06
*Pseudomonas aeruginosa*	8	4.1% (0.02–0.09)	0.00	85.25
*Enterococcus faecalis*	8	6.5% (0.11–0.30)	0.00	96.70
*Bacillus circulans*	6	3.7% (0.03–0.05)	0.53	0.00
*Enterococcus faecium*	6	7.0% (0.05–0.10)	0.34	12.20
*Pseudomonas gessardii*	7	5.0% (0.02–0.11)	0.00	80.29
*Pseudomonas psychrophila*	6	3.7% (0.01–0.11)	0.00	81.67
*Pseudomonas veronii*	6	1.8% (0.01–0.03)	0.89	0.00

## Data Availability

The original contributions presented in the study are included in the article. Further inquiries can be directed to the corresponding authors.

## Appendix A

**Table A1:** Summary of search queries.

Database	Search Query
CNKI	(Topic: Dairy Products) OR (Title/Abstract/Keyword: Dairy Products + Milk + Yogurt + Cheese + Butter + Milk Powder + Cream + Condensed Milk + Ice Cream + Dairy Beverage (Precise)) AND (Topic: Spoilage Bacteria) OR (Title/Abstract/Keyword: Spoilage Bacteria + Spoilage Microorganisms (Precise)) AND Publication Date Between (1 January 2003, 31 December 2025)
PubMed	((((((((“Dairy Products” [Mesh]) OR (Dairy Products [Title/Abstract])) OR (Dairy Product [Title/Abstract])) OR (Products, Dairy [Title/Abstract])) OR (Product, Dairy [Title/Abstract])) OR (Milk [Title/Abstract])) OR (Cheese [Title/Abstract])) OR (Yogurt [Title/Abstract])) AND (Spoilage Bacteria) AND ((“1 January 2003” [Date–Publication]: “31 December 2025” [Date–Publication]))
Web of Science	(TS = (Dairy Products) OR AB = (Dairy Products OR Dairy Product OR Products, Dairy OR Product, Dairy OR Milk OR Cheese OR Yogurt)) AND (TS = (Spoilage Bacteria) OR AB = (Spoilage Bacteria)) AND DOP = (1 January 2003/31 December 2025)

**Table A2:** The quality of Meta-analysis in article.

Number	NOS Evaluation Entry	Yes/Article (%)	No/Article (%)
1	Are the research objectives clearly defined?	79 (100.00%)	-
2	Do the samples meet the inclusion criteria?	79 (100.00%)	-
3	Do sampling locations be recorded?	79 (100.00%)	-
4	Has the sample size for the study been specified?	74 (93.67%)	5 (6.33%)
5	Are the sample types specified?	79 (100.00%)	-
6	Do the strain information be provided?	79 (100.00%)	-
7	Is standardized, credible authentication technology employed?	79 (100.00%)	-
8	Was appropriate statistical analysis employed?	79 (100.00%)	-
9	Are the results adequately presented?	79 (100.00%)	-

**Table A3:** Sample information collection form.

Sample Name	Positive Samples	Total Samples	Location	Testing Method	Research
Raw milk	8	8	United States	API 20 NE	[34]
Pasteurized milk	12	12	United States	API 50 CH and 16S rDNA	[35]
Raw milk	18	18	Belgium	16S rRNA	[36]
Cheese	15	50	Argentina	API 50 CH	[37]
Cheese	35	42	Sweden	16S rDNA	[38]
Raw milk	39	39	United States	16S rDNA	[39]
Pasteurized milk	11	11			
Cream	7	7	Japan	PCR-denaturing gradient gel electrophoresis	[40]
Raw milk	43	43	Brazil	API 50 CH and API Coryne	[41]
Refrigerated milk	40	40	Tunisia	API 20 NE	[42]
Cheese	16	16	Argentina	Bergey’s Manual	[43]
Pasteurized milk	29	95			
Yoghurt	4	4	China	API	[44]
Pasteurized milk	4	4			
Fluid milk	4	4			
Raw milk	8	8	Italy	RAPD-PCR	[45]
Pasteurized milk	5	5	United States	PCR-DGGE, 16S rRNA	[46]
Raw milk	62	62	Belgium	16S rRNA	[47]
Milk powder	92	100	Ireland	API 50 CHB	[48]
Cheese	/	140	Turkey	Gram staining	[49]
Cheese	2	2	United States	16S rDNA	[50]
Milk powder	22	22	Uruguay	16S rDNA	[51]
Milk powder	22	22	China	16S rDNA	[52]
Dried colostrum	7	7	United States	16S rRNA	[53]
Raw milk	201	1228	United States	16S rRNA	[54]
Pasteurized milk	1002	1228			
ESL milk	3	3	Germany	16S rRNA	[55]
Raw milk	/	3	Australia	16S rDNA	[56]
Pasteurized milk	/	3			
Cheese	/	3			
Milk powder	/	3			
Raw milk	1	1	Germany	FT-IR spectroscopy and partial gene sequencing	[57]
Cheese	2	5			
Yoghurt	2	3			
Milk powder	8	11			
UHT milk	0	2			
Pasteurized milk	3	4	Brazil	Bactray commercial kit	[58]
Raw milk	22	22	Spain	16S rRNA	[59]
Raw milk	/	/	China	16S rRNA	[60]
Raw milk	19	30	Tunisia	16S rRNA	[61]
UHT milk	10	30			
Pasteurized milk	11	20			
Raw milk	/	56	Italy	16S rRNA	[62]
Pasteurized milk	30	55	India	Selective medium culture combined with biochemical identification	[63]
Milk powder	18	52			
Ice cream	10	40			
Cheese	33	90			
Butter	5	25			
Raw milk	7	7	Australia	16S rRNA	[64]
Raw milk	3	3	Brazil	16S rDNA	[65]
Raw milk	203	211	Australia	The method of Manero and Blanch	[66]
Pasteurized milk	5	24			
Raw milk	20	20	Germany	16S rRNA	[67]
Raw milk	3	3	China	16S rDNA	[68]
Raw milk	61	61	Brazil	16S rRNA	[69]
Pasteurized milk	62	104	Australia	MALDI-TOF MS	[70]
Raw milk	92	154			
Raw milk	1	1	Slovakia	MALDI-TOF MS	[71]
Cheese	20	20	Italy	16S rRNA	[72]
Milk powder	6	6	New Zealand	16S rDNA	[73]
Raw milk	28	100	Tunisia	16S RNA	[74]
Bactofuged milk	10	100			
Pasteurized milk	16	100			
UHT milk	13	100			
Refrigerated milk	33	100			
Raw milk	10	10	United States	16S rRNA	[75]
Raw milk	93	120	United States	16S rRNA	[76]
Raw milk	14	30	Ghana	PCR	[77]
Cheese	10	31			
Yoghurt	12	28			
Raw milk	/	22	China	16S rRNA	[78]
Ice cream	38	125	Turkey	PCR	[79]
Milk powder	7	7	China	PCR	[80]
Raw milk	20	20	Brazil	PCR	[81]
Raw milk	20	20	Brazil	RFLP	[82]
Raw milk	20	20	Brazil	16S rRNA	[83]
Raw milk	46	125	China	16S rRNA	[84]
Raw milk	4	4	Malta	16S rRNA	[85]
Pasteurized milk	226	280	United States	16S rDNA	[86]
Raw milk	56	56	United States	16S rRNA	[87]
Cheese	2	2	Russia	DNA barcoding and DNA metabarcoding	[88]
Milk powder	2	3			
Yoghurt	2	3			
Milk powder	9	30	Poland	ISO 7932 [89]	[90]
Cheese	3	35			
Pasteurized milk	18	60			
Cheese	42	80			
Cheese	76	175			
Raw milk	4	4	Italy	16S rRNA	[91]
Pasteurized milk	2	8			
Cheese	3	20			
Raw milk	240	240	New Zealand	MALDI-TOF MS	[92]
Raw milk	50	50	Brazil	16S rRNA	[93]
Milk powder	21	21	France	16S rRNA	[94]
Cheese	89	340	Italy	ISO 7932:2005 [95]	[96]
Raw milk	27	27	Brazil	16S rRNA	[97]
Cream	64	64	France	Aamplified *panC* gene sequence analysis	[98]
Raw milk	6	6	China	Automatic microbial identification system	[99]
Milk powder	56	56	China	16S rRNA	[100]
UHT milk	53	100	Brazil	PCR	[101]
Cheese	32	32	Italy	MALDI-TOF MS	[102]
Raw milk	25	25	China	PCR	[14]
Raw milk	12	35	Turkey	PCR	[103]
Cheese	5	30			
Raw milk	3	3	Brazil	16S rRNA and 23S rRNA	[104]
Raw milk	100	100	Iran	16S rRNA	[105]
Yoghurt	12	12	Saudi Arabia	PCR	[106]
Cheese	2	2	China	16S rRNA	[107]
Raw milk	5	5	Brazil	*Pseudomonas* Agar Base supplemented with Cetrimide	[108]
Raw milk	50	50	Iraq	16S rRNA	[109]
Pasteurized milk	106	363	China	16S rDNA	[110]
Pasteurized milk	27	36	Canada	MALDI-TOF MS	[111]
Cheese	13	31			
Raw milk	21	21	Spain	PCR	[112]
Pasteurized milk	3	5			
Cheese	10	13			
Cream	2	2			
Raw milk	260	260	China	16S rRNA	[113]

**Table A4:** Strains information collection form.

Bacteria Strains	Positive Bacterial Strains	Total Bacterial Strains	Research
*Pseudomonas fluorescens*	172	338	[34]
*Pseudomonas putida*	47	338	
*Bacillus lentus*	1	53	[35]
*Bacillus cereus*	6	53	
*Bacillus licheniformis*	11	53	
*Bacillus mycoides*	1	53	
*Bacillus stearothermophilus*	3	53	
*Bacillus pumilus*	3	53	
*Microbacterium lacticum*	16	53	
*Kocuria varians*	4	53	
*Bacillus subtilis*	1	53	
*Bacillus barbaricus*	2	159	[36]
*Aneurinibacillus thermoaerophilus*	2	159	
*Bacillus smithii*	2	159	
*Bacillus fordii*	2	159	
*Virgibacillus proomii*	6	159	
*Brevibacillus agri*	8	159	
*Ureibacillus thermosphaericus*	11	159	
*Brevibacillus borstelensis*	12	159	
*Bacillus pallidus*	25	159	
*Bacillus fordii*	2	159	
*Bacillus licheniformis*	37	159	
*Bacillus subtilis*	2	159	
*Bacillus cereus*	15	50	[37]
*Bacillus pumilus*	20	50	
*Clostridium tyrobutyricum*	25	124	[38]
*Clostridium sporogenes*	40	124	
*Clostridium cochlearium*	15	124	
*Paenibacillus borealis*	2	12	[39]
*Paenibacillus amylolyticus*	1	12	
*Bacillus pumilus*	1	12	
*Bacillus weihenstephanensis*	1	12	
*Bacillus licheniformis*	1	12	
*Bacillus cereus*	1	12	[40]
*Bacillus amyloliquefaciens*	1	12	
*Bacillus subtilis*	1	12	
*Enterococcus faecium*	1	12	
*Enterococcus faecalis*	1	12	
*Lactobacillus paracasei*	1	12	
*Lactococcus lactis*	1	12	
*Leuconostoc mesenteroides*	1	12	
*Staphylococcus saprophyticus*	1	12	
*Staphylococcus warneri*	1	12	
*Achromobacter denitrificans*	1	12	
*Pseudomonas fluorescens*	1	12	
*Pseudomonas fluorescens*	94	250	[41]
*Pseudomonas putida*	3	250	
*Aeromonas caviae*	1	250	
*Aeromonas sobria*	1	250	
*Burkholderia cepacia*	12	250	
*Aeromonas hydrophila*	20	250	
*Klebsiella oxytoca*	10	250	
*Erwingella americana*	7	250	
*Chryseomonas luteola*	3	250	
*Alcaligenes faecalis*	1	250	
*Sphingomonas paucimobilis*	1	250	
*Bacillus coagulans*	1	250	
*Bacillus lentus*	2	250	
*Pseudomonas fluorescens*	52	258	[42]
*Aeromonas hydrophila*	41	258	
*Pseudomonas putida*	15	258	
*Pseudomonas cepacia*	34	258	
*Chryseomonas luteola*	13	258	
*Bacillus polymyxa*	3	17	[43]
*Bacillus macerans*	2	17	
*Leuconostoc mesenteroides*	5	17	
*Clostridium tyrobutyricum*	4	17	
*Lactobacillus fermentum*	3	17	
*Pseudomonas fluorescens*	1	3	[44]
*Kocuria varians*	1	3	
*Burkholderia cepacia*	1	3	
*Pseudomonas fluorescens*	1	66	[45]
*Serratia marcescens*	2	66	
*Klebsiella ornithionolytica*	1	66	
*Pseudomonas trivialis*	1	66	
*Pseudomonas reactans*	1	66	
*Pseudomonas putida*	1	66	
*Citrobacter freundii*	1	66	
*Pseudomonas fragi*	4	66	
*Pseudomonas lundensis*	1	66	
*Pseudomonas gessardii*	1	66	
*Hafnia alvei*	3	66	
*Staphylococcus pasteuri*	1	66	
*Bacillus thuringiensis*	1	66	
*Lactococcus lactis*	2	66	
*Corynebacterium variabilis*	1	66	
*Staphylococcus epidermidis*	1	66	
*Rodococcus erythropolis*	1	66	
*Carnobacterium piscicola*	1	66	
*Lactococcus piscium*	2	66	
*Staphylococcus warneri*	1	66	
*Pseudomonas migulae*	1	25	[46]
*Pseudomonas putida*	5	25	
*Pseudomonas fragi*	5	25	
*Pseudomonas fluorescens*	7	25	
*Clostridioides difficile*	1	25	
*Streptococcus agalactiae*	1	25	
*Buttiauxella izardii*	1	25	
*Buttiauxella brennerae*	1	25	
*Pseudomonas stutzeri*	1	25	
*Pseudomonas brassicacearum*	1	25	
*Buttiauxella warmboldiae*	1	25	
*Pseudomonas fragi*	25	103	[47]
*Pseudomonas lundensis*	68	103	
*Bacillus cereus*	59	100	[48]
*Bacillus licheniformis*	52	100	
*Bacillus subtilis*	38	100	
*Bacillus pumilus*	10	100	
*Bacillus mycoides*	3	100	
*Bacillus megaterium*	5	100	
*Bacillus polymyxa*	2	100	
*Bacillus sphaericus*	7	100	
*Bacillus circulans*	3	100	
*Bacillus coagulans*	3	100	
*Pseudomonas fluorescens*	1	140	[49]
*Pseudomonas pseudoalcaligenes*	22	140	
*Pseudomonas alcaligenes*	4	140	
*Pseudomonas aeruginosa*	1	140	
*Pseudomonas fluorescens*	17	26	[50]
*Bacillus cereus*	1	26	
*Lactococcus lactis*	8	26	
*Bacillus licheniformis*	88	601	[51]
*Bacillus megaterium*	10	207	
*Bacillus pumilus*	6	207	
*Anoxybacillus flavithermus*	86	207	
*Bacillus subtilis*	10	207	
*Bacillus licheniformis*	223	801	[52]
*Anoxybacillus flavithermus*	164	801	
*Geobacillus stearothermophilus*	99	801	
*Bacillus lichenformis*	5	29	[53]
*Bacillus badius*	2	29	
*Kocuria rhizophila*	2	29	
*Bacillus cereus*	2	29	
*Bacillus subtilis*	9	29	
*Pseudomonas fluorescens*	4	29	
*Enterococcus faecium*	1	29	
*Bacillus massiliensis*	1	29	
*Microbacterium hydrocarbonoxydans*	1	29	
*Bacillus pumilus*	108	1288	[54]
*Bacillus licheniformis*	188	1288	
*Bacillus subtilis*	23	1288	
*Bacillus cereus*	27	1288	
*Paenibacillus odorifer*	506	1288	
*Paenibacillus graminis*	46	1288	
*Paenibacillus amylolyticus*	101	1288	
*Bacillus cereus*	12	162	[55]
*Microbacterium lacticum*	26	162	
*Bacillus subtilis*	29	162	
*Bacillus licheniformis*	2	162	
*Bacillus pumilus*	48	162	
*Bacillus circulans*	2	162	
*Lactococcus lactis*	1	162	
*Enterococcus casseliflavus*	1	162	
*Acinetobacter johnsonii*	2	162	
*Chryseobacterium haifense*	2	162	
*Paenibacillus borealis*	1	162	
*Bacillus flexus*	1	162	
*Brevibacillus agri*	8	162	
*Lysinibacillus fusiformis*	3	162	
*Paenibacillus turicensis*	1	162	
*Moraxella osloensis*	2	162	
*Psychrobacter glacincola*	2	162	
*Streptococcus thermophilus*	19	162	
*Bacillus licheniformis*	60	237	[56]
*Bacillus subtilis*	25	237	
*Bacillus pumilus*	20	237	
*Bacillus cereus*	20	237	
*Anoxybacillus flavithermus*	19	237	
*Paenibacillus polymyxa*	1	237	
*Bacillus megaterium*	8	237	
*Bacillus clausii*	1	237	
*Bacillus subtilis*	38	379	[57]
*Bacillus licheniformis*	72	379	
*Geobacillus stearothermophilus*	30	379	
*Bacillus cereus*	88	379	
*Bacillus circulans*	18	379	
*Bacillus pumilus*	13	379	
*Pseudomonas fluorescens*	12	100	[58]
*Pseudomonas aeruginosa*	4	100	
*Pseudomonas mendocina*	1	100	
*Pseudomonas pseudoalcaligenes*	1	100	
*Streptococcus thermophilus*	10	164	[59]
*Lactococcus lactis*	115	164	
*Lactococcus garvieae*	28	164	
*Enterococcus durans*	1	164	
*Enterococcus faecalis*	2	164	
*Streptococcus agalactiae*	6	164	
*Lactococcus raffinolactis*	4	164	
*Leuconostoc pseudomesenteroides*	1	164	
*Streptococcus salivarius*	1	164	
*Bacillus subtilis*	1	5	[60]
*Bacillus licheniformis*	1	5	
*Bacillus stearothermophilus*	1	5	
*Bacillus sporothermodurans*	25	100	[61]
*Bacillus cereus*	22.5	100	
*Bacillus subtilis*	12.5	100	
*Bacillus licheniformis*	12.5	100	
*Bacillus pumilus*	10	100	
*Brevibacillus brevis*	10	100	
*Bacillus sphaericus*	7.5	100	
*Pseudomonas fluorescens*	85	178	[108]
*Pseudomonas aeruginosa*	13	178	
*Pseudomonas putida*	34	178	
*Pseudomonas fluorescens*	19	80	[62]
*Pseudomonas aeruginosa*	15	80	
*Pseudomonas fragi*	3	80	
*Pseudomonas putida*	7	80	
*Pseudomonas rhodesiae*	2	80	
*Pseudomonas fulva*	4	80	
*Pseudomonas libanensis*	1	80	
*Hafnia alvei*	5	80	
*Serratia marcescen*	9	80	
*Bacillus cereus*	55	100	[63]
*Bacillus cereus*	33	100	
*Pseudomonas fluorescens*	4	58	[64]
*Pseudomonas lurida*	1	58	
*Pseudomonas gessardii*	3	58	
*Pseudomonas proteolytica*	1	58	
*Pseudomonas gingeri*	1	58	
*Pseudomonas poae*	3	58	
*Pseudomonas veronii*	1	58	
*Pseudomonas valomonii*	1	58	
*Pseudomonas psychrophila*	2	58	
*Pseudomonas fragi*	4	58	
*Bacillus pumilus*	2	58	
*Bacillus licheniformis*	5	58	
*Bacillus safensis*	1	58	
*Hafnia alvei*	5	58	
*Hafnia paralvei*	2	58	
*Bacillus weihenstephanensis*	3	58	
*Acinetobacter guillouiae*	1	58	
*Serratia liquefaciens*	1	58	
*Stenotrophomonas maltophilia*	1	58	
*Pseudomonas lundensis*	2	58	
*Pseudomonas fluorescens*	4	8	[65]
*Pseudomonas putida*	2	8	
*Serratia liquefaciens*	2	8	
*Enterococcus casseliflavus*	7	211	[66]
*Enterococcus faecalis*	157	211	
*Enterococcus faecium*	18	211	
*Enterococcus durans*	6	211	
*Enterococcus asini*	2	211	
*Enterococcus malodoratus*	1	211	
*Enterococcus gallinarum*	1	211	
*Enterococcus mundtii*	1	211	
*Enterococcus pseudoavium*	1	211	
*Enterococcus raffinosus*	1	211	
*Enterococcus sulfureus*	1	211	
*Enterococcus saccharolyticus*	1	211	
*Enterococcus hirae*	18	211	
*Lactococcus lactis*	11	100	[67]
*Pseudomonas proteolytica*	9	100	
*Pseudomonas fragi*	4	100	
*Pseudomonas lundensis*	8	100	
*Lactococcus raffinolactis*	3	100	
*Pseudomonas putida*	1	34	[68]
*Enterobacter ludwigii*	1	34	
*Citrobacter freundii*	1	34	
*Kurthia pneumonia*	1	34	
*Kurthia gibsonii*	2	34	
*Lactococcus garvieae*	1	34	
*Lactococcus lactis*	2	34	
*Acinetobacter lwoffii*	1	34	
*Enterococcus faecalis*	1	34	
*Pseudomonas aeruginosa*	1	34	
*Pseudomonas fluorescens*	41	82	[69]
*Acinetobacter guillouiae*	4	697	[70]
*Buttiauxella agrestis*	5	697	
*Buttiauxella gaviniae*	6	697	
*Buttiauxella noackiae*	7	697	
*Citrobacter braakii*	23	697	
*Citrobacter freundii*	15	697	
*Escherichia coli*	121	697	
*Enterobacter amnigenus*	24	697	
*Enterobacter asburiae*	43	697	
*Enterobacter cloacae*	52	697	
*Enterobacter kobei*	7	697	
*Enterobacter ludwigii*	15	697	
*Ewingella americana*	5	697	
*Hafnia alvei*	52	697	
*Klebsiella oxytoca*	70	697	
*Klebsiella pneumonia*	16	697	
*Kluyvera intermedia*	7	697	
*Pantoea agglomerans*	9	697	
*Proteus mirabilis*	2	697	
*Proteus vulgaris*	2	697	
*Pseudomonas aeruginosa*	2	697	
*Pseudomonas fluorescens*	4	697	
*Pseudomonas lundensis*	2	697	
*Pseudomonas nitroreducens*	2	697	
*Rahnella aquatilis*	9	697	
*Raoultella ornithinolytica*	114	697	
*Raoultella terrigena*	3	697	
*Serratia fonticolla*	2	697	
*Serratia liquefaciens*	48	697	
*Serratia marcescens*	11	697	
*Stenotrophomonas maltophilia*	2	697	
*Yersinia enterocolitica*	12	697	
*Pseudomonas fragi*	30	200	[71]
*Pseudomonas gessardii*	19	200	
*Pseudomonas lundensis*	34	200	
*Acinetobacter johnsonii*	11	200	
*Sphingobacterium multivorum*	3	200	
*Pseudomonas fragi*	20	200	
*Raoultella ornithinolytica*	17	200	
*Corynebacterium casei*	17	200	
*Lactococcus lactis*	15	200	
*Aeromonas media*	9	200	
*Kytococcus sedentarius*	6	200	
*Citrobacter braakii*	6	200	
*Enterococcus faecalis*	1	200	
*Candida inconspicua*	3	200	
*Bacillus muralis*	38	188	[72]
*Clostridium butyricum*	6	188	
*Lactobacillus helveticus*	1	188	
*Lactobacillus zeae*	1	188	
*Paenibacillus amylolyticus*	29	188	
*Staphylococcus equorum*	1	188	
*Streptococcus infantis*	1	188	
*Acinetobacter johnsonii*	3	188	
*Acinetobacter iwofii*	1	188	
*Azomonas insignis*	1	188	
*Enhydrobacter aerosaccus*	1	188	
*Hemophilus parainfluenza*	1	188	
*Propionibacterium acnes*	1	188	
*Pseudomonas fragi*	2	188	
*Bacillus cereus*	22	188	
*Pseudomonas fragi*	3	188	
*Pseudomonas veronii*	2	188	
*Sphingomonas echinoides*	1	188	
*Anoxybacillus kestabolensis*	1	188	
*Bacillus anthracis*	1	188	
*Bacillus flexus*	1	188	
*Bacillus horikoshii*	1	188	
*Clostridium bowmanii*	11	188	
*Clostridium butyricum*	5	188	
*Clostridium neonatale*	3	188	
*Clostridium pasteurianum*	38	188	
*Clostridium tyrobutiricum*	1	188	
*Lysinibacillus boronitolerans*	6	188	
*Paenibacillus lentimorbus*	2	188	
*Paenibacillus stellifer*	1	188	
*Hemophilus parainfluenzae*	1	188	
*Neisseria subflava*	1	188	
*Bacillus licheniformis*	67	100	[73]
*Bacillus cereus*	19	100	
*Bacillus thuringiensis*	4	100	
*Bacillus subtilis*	4	100	
*Bacillus pumilus*	4	100	
*Paenibacillus glucanolyticus*	2	100	
*Bacillus pumilus*	9.52	100	[74]
*Bacillus licheniformis*	52.38	100	
*Terribacillus aidingensis*	4.76	100	
*Bacillus sporothermodurans*	4.76	100	
*Bacillus cereus*	29	1000	[75]
*Bacillus subtilis*	191	1000	
*Bacillus licheniformis*	323	1000	
*Bacillus safensis*	59	1000	
*Bacillus aerophilus*	59	1000	
*Bacillus odorifer*	44	1000	
*Bacillus aerophilus*	8	106	[76]
*Bacillus cereus*	3	106	
*Bacillus clausii*	1	106	
*Bacillus horneckiae*	1	106	
*Bacillus licheniformis*	34	106	
*Bacillus pumilus*	36	106	
*Bacillus safensis*	6	106	
*Bacillus subtilis*	9	106	
*Bacillus weihenstephanensis*	1	106	
*Paenibacillus amylolyticus*	1	106	
*Paenibacillus cookii*	2	106	
*Paenibacillus peoriae*	3	106	
*Bacillus cereus*	36	89	[77]
*Pseudomonas fragi*	9	21	[78]
*Pseudomonas putida*	2	21	
*Pseudomonas prosekii*	1	21	
*Pseudomonas fluorescens*	6	21	
*Pseudomonas psychrophila*	3	21	
*Bacillus cereus*	38	125	[79]
*Bacillus cereus*	31.82	100	[80]
*Lactococcus lactis*	5.09	100	
*Alkaliphilus oremlandii*	4.17	100	
*Cronobacter sakazakii*	3.76	100	
*Bacillus circulans*	3.39	100	
*Acinetobacter johnsonii*	2.41	100	
*Paenibacillus barcinonensis*	1.9	100	
*Paenibacillus amylolyticus*	1.53	100	
*Paenibacillus odorifer*	1.08	100	
*Lactococcus lactis*	35	141	[81]
*Enterobacter kobei*	22	141	
*Aerococcus urinaeequi*	12	141	
*Acinetobacter lwoffii*	9	141	
*Kurthia gibsonii*	8	141	
*Serratia ureilytica*	8	141	
*Bacillus licheniformis*	7	141	
*Staphylococcus epidermidis*	7	141	
*Macrococcus caseolyticus*	6	141	
*Streptococcus parauberis*	5	141	
*Enterococcus faecium*	4	141	
*Acinetobacter johnsonii*	3	141	
*Enterococcus thailandicus*	2	141	
*Stenotrophomonas rhizophila*	2	141	
*Yersinia enterocolitica*	2	141	
*Acinetobacter bouvetii*	1	141	
*Bacillus cereus*	1	141	
*Bacillus pumilus*	1	141	
*Enterococcus hermanniensis*	1	141	
*Staphylococcus warneri*	1	141	
*Bacillus licheniformis*	45	132	[82]
*Brachybacterium nesterenkovii*	19	132	
*Enterococcus faecalis*	12	132	
*Streptococcus thermophilus*	11	132	
*Micrococcus aloeverae*	6	132	
*Streptococcus thermophilus*	6	132	
*Kocuria varians*	5	132	
*Bacillus circulans*	4	132	
*Paenibacillus cookii*	4	132	
*Kocuria kristinae*	3	132	
*Bacillus clausii*	2	132	
*Bacillus invictae*	2	132	
*Paenibacillus puldeungensis*	2	132	
*Streptococcus gallolyticus*	2	132	
*Macrococcus carouselicus*	1	132	
*Paenibacillus wynnii*	1	132	
*Bacillus licheniformis*	22	40	[83]
*Bacillus pumilus*	2	40	
*Bacillus circulans*	1	40	
*Pseudomonas putida*	11	67	[84]
*Pseudomonas fluorescens*	21	67	
*Pseudomonas fragi*	11	67	
*Pseudomonas moraviensis*	1	67	
*Pseudomonas rhodesiae*	1	67	
*Pseudomonas protegens*	1	67	
*Pseudomonas lurida*	1	67	
*Pseudomonas reactans*	1	67	
*Pseudomonas chlororaphis*	1	67	
*Pseudomonas poae*	1	67	
*Pseudomonas chlororaphis*	1	67	
*Pseudomonas koreensis*	3	67	
*Pseudomonas graminis*	2	67	
*Pseudomonas psychrophila*	1	67	
*Pseudomonas veronii*	1	67	
*Pseudomonas reinekei*	1	67	
*Pseudomonas cedrina*	1	67	
*Microbacterium resistens*	1	30	[85]
*Rhodococcus pyridinivorans*	3	30	
*Bacillus cereus*	7	30	
*Enterococcus faecalis*	3	30	
*Enterococcus faecium*	2	30	
*Cellulosimicrobium funkei*	2	30	
*Brachybacterium nesterenkovii*	1	30	
*Streptococcus macedonicus*	1	30	
*Sphingobacterium arenae*	1	30	
*Gordonia sputi*	1	30	
*Pseudomonas fluorescens*	92	148	[86]
*Acinetobacter guillouiae*	6	148	
*Acinetobacter haemolyticus*	8	148	
*Comamonas thiooxydans*	3	148	
*Enterobacter ludwigii*	2	148	
*Hafnia paralvei*	2	148	
*Janthinobacterium lividum*	2	148	
*Lelliottia amnigena*	5	148	
*Limnohabitans planktonicus*	3	148	
*Pantoea anthophila*	2	148	
*Pseudomonas putida*	4	148	
*Rahnella aquatilis*	2	148	
*Stenotrophomonas rhizophila*	11	148	
*Pseudomonas azotoformans*	8	54	[87]
*Pseudomonas aeruginosa*	5	54	
*Pseudomonas koreensis*	4	54	
*Pseudomonas gessardii*	3	54	
*Pseudomonas lurida*	3	54	
*Pseudomonas monteilii*	3	54	
*Pseudomonas brenneri*	2	54	
*Pseudomonas lini*	2	54	
*Pseudomonas kilonensis*	2	54	
*Pseudomonas mucidolens*	2	54	
*Pseudomonas proteolytica*	2	54	
*Pseudomonas veronii*	2	54	
*Pseudomonas antarctica*	1	54	
*Pseudomonas chlororaphis*	1	54	
*Pseudomonas congelans*	1	54	
*Pseudomonas ficuserectae*	1	54	
*Pseudomonas fluorescens*	1	54	
*Pseudomonas jessenii*	1	54	
*Pseudomonas lundensis*	1	54	
*Pseudomonas mediterranea*	1	54	
*Pseudomonas migulae*	1	54	
*Pseudomonas panacis*	1	54	
*Pseudomonas plecoglossicida*	1	54	
*Pseudomonas salomonii*	1	54	
*Pseudomonas simiae*	1	54	
*Pseudomonas taetrolens*	1	54	
*Pseudomonas trivialis*	1	54	
*Pseudomonas Vancouverensis*	1	54	
*Pseudomonas fluorescens*	3	19	[88]
*Staphylococcus epidermidis*	1	19	
*Lactococcus lactis*	2	19	
*Moraxella osloensis*	1	19	
*Kocuria rosea*	1	19	
*Klebsiella pneumoniae*	1	19	
*Staphylococcus warneri*	1	19	
*Klebsiella pneumonia*	1	19	
*Bacillus cereus*	148	380	[90]
*Pseudomonas fluorescens*	105.6	300	[91]
*Pseudomonas putida*	127.2	300	
*Pseudomonas aeruginosa*	13.3	300	
*Pseudomonas oleovorans*	6.7	300	
*Pseudomonas anguilliseptica*	2.2	300	
*Pseudomonas lundensis*	24.41	100	[92]
*Pseudomonas fragi*	20.47	100	
*Pseudomonas fluorescens*	5.51	100	
*Pseudomonas taetrolens*	2.36	100	
*Pseudomonas synxantha*	0.79	100	
*Pseudomonas rhodesiae*	3.15	100	
*Pseudomonas protegens*	0.79	100	
*Pseudomonas libanensis*	0.79	100	
*Pseudomonas koreensis*	2.36	100	
*Pseudomonas gessardii*	2.36	100	
*Pseudomonas extremorientalis*	1.57	100	
*Pseudomonas antarctica*	0.79	100	
*Rahnella aquatillis*	0.79	100	
*Hafnia alvei*	0.79	100	
*Lactococcus raffinolactis*	0.79	100	
*Carnobacterium maltaromaticum*	0.79	100	
*Buttiauxella brennerae*	0.79	100	
*Bacillus cereus*	0.79	100	
*Acinetobacter guillouiae*	0.79	100	
*Pseudomonas fluorescens*	23.1	100	[93]
*Bacillus subtilis*	23	139	[94]
*Bacillus pumilis*	7	139	
*Bacillus amyloliquefaciens*	23	139	
*Bacillus licheniformis*	16	139	
*Bacillus coagulans*	1	139	
*Bacillus badius*	1	139	
*Bacillus cereus*	51	139	
*Geobacillus toebii*	3	139	
*Bacillus cereus*	89	340	[96]
*Lysinibacillus fusiformis*	80	229	[97]
*Macrococcus caseolyticus*	22	229	
*Micrococcus aloeverae*	22	229	
*Bacillus licheniformis*	15	229	
*Lysinibacillus varians*	15	229	
*Bacillus toyonensis*	14	229	
*Aestuariimicrobium kwangyangense*	12	229	
*Carnobacterium divergens*	12	229	
*Microbacterium hominis*	9	229	
*Microbacterium lacticum*	8	229	
*Kocuria indica*	6	229	
*Micrococcus yunnanensis*	6	229	
*Bacillus invictae*	3	229	
*Gordonia paraffinivorans*	3	229	
*Kocuria indica*	2	229	
*Bacillus cereus*	52	81	[98]
*Bacillus flexus*	1	22	[100]
*Bacillus thuringiensis*	1	22	
*Bacillus licheniformis*	5	22	
*Bacillus cereus*	13	22	
*Streptococcus agalactiae*	4	29	[99]
*Geobacillus stearothermophilus*	2	29	
*Bacillus megaterium*	3	29	
*Bacillus subtilis*	3	29	
*Acinetobacter lwoffii*	3	29	
*Bacillus cereus*	69	239	[101]
*Bacillus cereus*	72	103	[102]
*Bacillus subtilis*	31	103	
*Pseudomonas fluorescens*	88	116	[14]
*Pseudomonas veronii*	2	116	
*Pseudomonas psychrophila*	1	116	
*Pseudomonas lundensis*	8	116	
*Pseudomonas lactis*	2	116	
*Pseudomonas azotoformans*	3	116	
*Pseudomonas granadensis*	4	116	
*Pseudomonas lurida*	3	116	
*Pseudomonas rhizosphaerae*	1	116	
*Pseudomonas extremorientalis*	2	116	
*Pseudomonas rhodesiae*	2	116	
*Bacillus cereus*	12	35	[103]
*Bacillus cereus*	5	30	
*Bacillus toyonensis*	66	107	[104]
*Bacillus licheniformis*	11	107	
*Lysinibacillus fusiformis*	20	107	
*Bacillus pumilus*	3	107	
*Bacillus flexus*	3	107	
*Bacillus invictae*	2	107	
*Bacillus megaterium*	2	107	
*Pediococcus pentosaceus*	1	36	[105]
*Lactococcus lactis*	2	36	
*Enterococcus faecium*	4	36	
*Pediococcus acidilactici*	1	36	
*Enterococcus faecalis*	2	36	
*Levilactobacillus brevis*	1	36	
*Staphylococcus aureus*	1	36	
*Lactococcus garvieae*	2	36	
*Bacillus licheniformis*	2	36	
*Pediococcus acidilactici*	2	36	
*Staphylococcus epidermidis*	2	36	
*Streptococcus pyogenes*	1	36	
*Lactiplantibacillus plantarum*	1	36	
*Bacillus cereus*	1	36	
*Staphylococcus aureus*	1	36	
*Staphylococcus hyicus*	2	36	
*Staphylococcus haemolyticus*	1	36	
*Bacillus mycoides*	1	36	
*Weizmannia coagulans*	2	36	
*Microbacterium lacticum*	2	36	
*Bacillus subtilis*	1	36	
*Bacillus thuringiensis*	1	36	
*Paenibacillus polymyxa*	1	36	
*Leuconostoc mesenteroides*	1	36	
*Bacillus cereus*	52	131	[106]
*Bacillus subtilis*	26	131	
*Serratia marcescens*	23	131	
*Bacillus subtilis*	3	8	[107]
*Bacillus velezensis*	5	8	
*Pseudomonas fluorescens*	19	50	[109]
*Pseudomonas lurida*	31	106	[110]
*Pseudomonas gessardii*	20	106	
*Pseudomonas psychrophile*	16	106	
*Pseudomonas lundensis*	6	106	
*Pseudomonas qingdaonensis*	6	106	
*Microbacterium lacticum*	1	31	[111]
*Paenibacillus odorifer*	1	31	
*Lactococcus raffinolactis*	5	31	
*Pseudomonas libanensis*	1	31	
*Bacillus licheniformis*	1	31	
*Sphingobium yanoikuyae*	1	31	
*Bacillus cereus*	1	31	
*Staphylococcus aureus*	1	31	
*Lactococcus lactis*	4	31	
*Staphylococcus warneri*	1	31	
*Klebsiella pneumoniae*	1	31	
*Pseudomonas qingdaonensis*	2	31	
*Microbacterium lacticum*	1	31	
*Paenibacillus odorifer*	1	31	
*Lactococcus raffinolactis*	1	31	
*Pseudomonas libanensis*	1	31	
*Bacillus licheniformis*	3	31	
*Sphingobium yanoikuyae*	1	31	
*Bacillus cereus*	1	31	
*Staphylococcus aureus*	1	31	
*Lactococcus lactis*	1	31	
*Pseudomonas putida*	2	208	[112]
*Pseudomonas atacamensis*	6	208	
*Pseudomonas fragi*	21	208	
*Pseudomonas fluorescens*	34	208	
*Pseudomonas lundensis*	21	208	
*Pseudomonas shahriarae*	11	208	
*Pseudomonas koreensis*	7	208	
*Pseudomonas salmasensis*	4	208	
*Pseudomonas canadensis*	4	208	
*Pseudomonas veronii*	4	208	
*Pseudomonas weihenstephanensis*	3	208	
*Pseudomonas putida*	1	208	
*Pseudomonas atacamensis*	2	208	
*Pseudomonas fragi*	1	208	
*Pseudomonas fluorescens*	1	208	
*Pseudomonas lundensis*	1	208	
*Pseudomonas shahriarae*	2	208	
*Pseudomonas koreensis*	1	208	
*Pseudomonas salmasensis*	1	208	
*Pseudomonas canadensis*	1	208	
*Pseudomonas veronii*	2	208	
*Pseudomonas fluorescens*	121	1430	[113]
*Acinetobacter junii*	73	1430	
*Pseudomonas brenneri*	68	1430	

**Table A5:** Publication bias of the literature.

Analysis Type	Number of Studies (k)	Threshold (5k + 10)	Fail-Safe N Analysis	Egger’s Regression Test (*p*)
The overall prevalence	79	405	1664	0.00
Prevalence of *Bacillus cereus*	35	185	6659	0.13
Prevalence of *Bacillus licheniformis*	27	145	6450	0.34
Prevalence of *Pseudomonas fluorescens*	30	160	3361	0.37
Prevalence of *Bacillus subtilis*	21	115	4857	0.21
Prevalence of *Bacillus pumilus*	18	100	3877	0.30
Prevalence of *Pseudomonas fragi*	14	80	1733	0.05
Prevalence of *Pseudomonas putida*	14	80	1613	0.01
Prevalence of *Lactococcus lactis*	13	75	671	0.01
Prevalence of *Pseudomonas lundensis*	11	65	957	0.03
Prevalence of *Pseudomonas aeruginosa*	8	50	713	0.10
Prevalence of *Enterococcus faecalis*	8	50	162	0.01
Prevalence of *Bacillus circulans*	6	40	432	0.03
Prevalence of *Enterococcus faecium*	6	40	224	0.47
Prevalence of *Pseudomonas gessardii*	7	45	410	0.05
Prevalence of *Pseudomonas psychrophila*	6	40	218	0.03
Prevalence of *Pseudomonas veronii*	6	40	270	0.79

**Table A6:** Characteristics of lipases from spoilage bacteria.

Host Strain	GenBank	Conservative Domain	Length /a.a	Weight /kDa	PI	Net Negative Charge	Net Positive Charge	Molar Absorptivity	Fat Coefficient	Average Hydrophilicity	Instability Index
*Bacillus cereus*	WCT66113.1	Abhydrolase super family	527	58.48	4.94	84	57	0.809	93.83	−0.306	23.42
	ETT75475.1	EstA super family	413	46.09	6.95	38	37	1.984	79.08	−0.355	26.43
	ETT75504.1	MenH super family/Abhydrolase super family	277	32.78	5.36	45	33	2.314	86.53	−0.481	40.24
	RXG07039.1	SGNH_hydrolase super family	293	32.97	8.77	35	39	1.213	94.4	−0.273	26.95
	ALZ63309.1	CusA super family/Aes super family	402	44.97	6.38	40	37	1.786	100.87	−0.018	37.22
	EOP28808.1	MenH super family	363	41.94	7.73	44	45	0.905	87.22	−0.287	34.83
	EOP56421.1	EstA super family/Abhydrolase super family	534	58.91	8.12	54	56	1.568	79.94	−0.321	31.69
*Bacillus licheniformis*	WKU31742.1	CVT17 super family	479	53.95	5.85	71	63	1.2	76.97	−0.741	22.19
	TWO06542.1	EstA super family	487	53.67	8.98	48	54	1.292	77.97	−0.351	25.25
	ARW52538.1	SGNH_hydrolase super family	212	24.12	5.54	33	28	0.851	90.14	−0.378	34.4
	VEH78556.1	SGNH_hydrolase super family	250	28.47	4.97	44	34	0.862	87.72	−0.416	35.75
*Bacillus subtilis*	CUB56573.1	Abhydrolase super family/CVT17 super family	416	47.24	5.82	66	57	1.118	69.11	−0.786	33.48
	WP_251187522.1	CVT17 super family	487	55.15	5.63	73	57	0.794	84.5	−0.597	30.83
	CON51271.1	DAP2 super family	257	29.74	9.68	26	36	1.291	73.54	−0.506	52.57
	KAF2426737.1	Lip2 super family	276	31.23	8.97	22	29	0.871	72.5	−0.226	40.41
*Bacillus pumilus*	OUZ06619.1	CVT17 superfamily	496	55.82	8.47	65	68	0.864	82.96	−0.55	40.05
*Pseudomonas fluorescens*	POH41489.1	Abhydrolase super family	716	81.58	7.23	83	82	1.086	78.39	−0.468	50.58
	VEF10173.1	COG4757 super family	329	36.59	7.15	36	36	2.17	82.19	−0.29	42.06
	ROO16718.1	Lip2 super family	776	86.82	6.05	93	76	1.2	79.96	−0.4	47.75
*Pseudomonas fragi*	ARQ76605.1	Abhydrolase super family	676	75.62	6.6	74	68	1.006	82.69	−0.281	48.5
	VFB20546.1	Abhydrolase super family/HemolysinCabind super family/COG2931 super family	616	64.93	4.65	71	36	1.244	82.86	−0.17	26.01
*Pseudomonas putida*	ANC02924.1	Abhydrolase super family	328	35.87	5.06	43	26	1.454	92.29	−0.1	56.56
	SUD66897.1	PldB super family	338	37.99	8.82	28	31	1.922	98.17	−0.063	54.12
	BAW21301.1	COG4782 super family	329	36.56	8.61	40	44	1.43	83.95	−0.48	30.04
*Pseudomonas lundensis*	AOZ11414.1	Axe1 super family/Abhydrolase super family	320	35.04	4.92	43	25	1.413	92.72	−0.027	46.82
*Enterococcus faecalis*	BDQ58792.1	Aes super family	337	37.85	8.69	30	34	1.356	85.64	−0.167	34.34
	GEJ62098.1	CwlO1 super family/CHAP super family	482	50.9	5.02	63	51	0.882	71.37	−0.53	40.43
	VTT41658.1	CVT17 super family	465	52.53	5.21	63	50	1.261	79.91	−0.535	28.71
*Enterococcus faecium*	OAQ37576.1	Abhydrolase super family	315	35.58	4.89	49	34	1.275	86.41	−0.357	40.67
	GJG91484.1	Aes super family	337	38.33	6.26	42	40	1.238	79.88	−0.383	30.72
	XLE04444.1	CVT17 super family	418	46.97	6.96	56	56	1.083	85.93	−0.47	23.8
*Lactococcus lactis*	WP_109991484.1	Abhydrolase super family	415	45.63	6.11	49	45	1.323	77.54	−0.462	23.53
	KRO23992.1	FrmB super family	258	29.67	5.34	32	25	2.151	79.77	−0.359	34.57
	AIS02893.1	KxYKxGKxW_sig superfamily/Abhydrolase super family	561	58.5	4.1	38	14	1.048	92.39	0.082	37.18
	PFG86633.1	SGNH_hydrolase super family	428	46.69	9.22	39	46	1.443	86.8	−0.264	23.34
	SPS11769.1	Aes super family/DAP2 super family	257	28.84	5.79	32	28	1.769	94.94	−0.342	26.13
	BFW28476.1	SGNH_hydrolase super family	224	25.81	4.85	36	22	0.733	82.72	−0.515	34.14
